# Calcinosis in juvenile dermatomyositis: Updates on pathogenesis and treatment

**DOI:** 10.3389/fmed.2023.1155839

**Published:** 2023-03-02

**Authors:** Caitlan S. Pinotti, Laura Cannon, Jeffrey A. Dvergsten, Eveline Y. Wu

**Affiliations:** ^1^Division of Pediatric Rheumatology, Department of Pediatrics, Duke University, Durham, NC, United States; ^2^Division of Pediatric Rheumatology, Department of Pediatrics, University of North Carolina, Chapel Hill, Chapel Hill, NC, United States; ^3^Division of Pediatric Allergy and Immunology, Department of Pediatrics, University of North Carolina Chapel Hill, Chapel Hill, NC, United States

**Keywords:** juvenile dermatomyositis, calcinosis, myositis-specific antibody, pathogenesis, treatment

## Abstract

Calcinosis, or the deposition of insoluble calcium salts in the skin, subcutaneous tissue, fascia, tendons, and muscles, is a feared complication of juvenile dermatomyositis. Calcinosis is estimated to affect up to 40% of patients with juvenile dermatomyositis and contributes to significant disease morbidity. Calcinosis can be challenging to treat, and the most effective treatment remains unknown because of a lack of comparative studies. We aim to review the literature published in the last 5 years to summarize updates on the pathogenesis and treatment of calcinosis in juvenile dermatomyositis and describe future areas for research.

## Introduction

**FIGURE 1:**
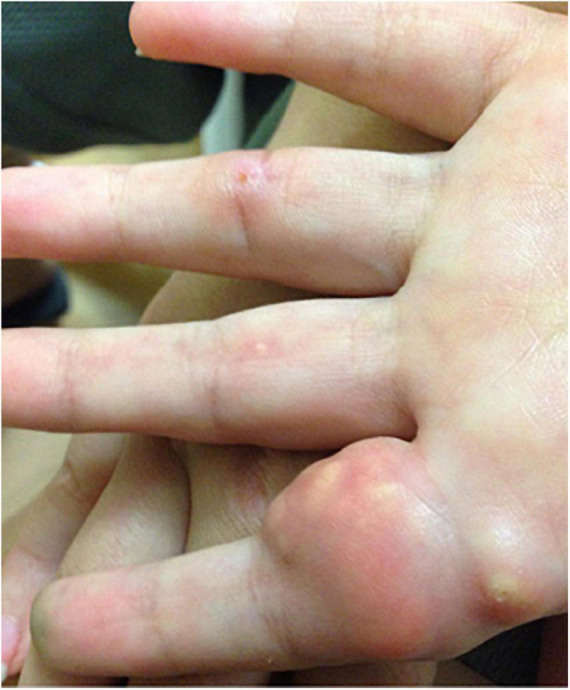
Photograph of calcinosis lesions of the fingers of a child with juvenile dermatomyositis. Permission obtained from the patient for publication of this image.

**FIGURE 2:**
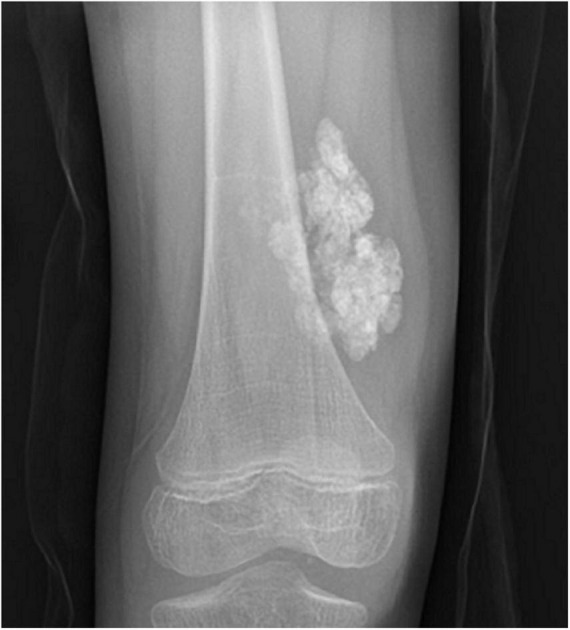
Plain radiograph of calcinosis lesions in the subcutaneous tissues adjacent to the right knee of a child with juvenile dermatomyositis. Permission obtained from the patient for publication of this image.

Juvenile dermatomyositis (JDM) is the most common inflammatory myopathy in children, affecting 2-4 children per million per year (1). Children typically present with proximal muscle weakness and pathognomonic rashes, notably Gottron’s sign and heliotrope (2). Despite improvements in mortality, significant morbidity associated with JDM remains. Calcinosis, which is the intracellular deposition of insoluble calcium salts in the skin, subcutaneous tissue, fascia, tendons, and muscles, affects approximately 40% of patients and can lead to skin ulcers and recurrent infection, joint contractures, and nerve entrapment. Calcinosis in JDM is dystrophic, occurring secondary to injury and in the setting of otherwise normal calcium and phosphate (3). Figure 1 shows calcinosis in the fingers of a child with JDM, and Figure 2 is a radiograph of calcinosis in the subcutaneous tissues near the knee. Prior studies have suggested that risk factors include delay in JDM diagnosis and prolonged disease activity, implying that early and aggressive treatment may minimize calcinosis risk (4). There remains a lack of consensus on appropriate treatment of calcinosis in JDM, likely due to an unclear understanding of its pathogenesis, disease rarity, and reliance on expert opinion *via* case reports and case series. This article is a review of literature published in the past 5 years regarding calcinosis in JDM, addressing updates in pathogenesis and treatment.

## Pathogenesis

A 2022 review by Davuluri et al. (5) summarizes potential pathogenic mechanisms of calcinosis in JDM, including the process of mitochondrial calcification (3) which is driven primarily by reactive oxygen species (ROS) produced secondary to inflammation. Calcified mitochondria have been found in skeletal muscle cells of JDM patients (6), and once calcified, the mitochondria perpetuate inflammation by secreting inflammatory cytokines like IL-1β and upregulating type I interferon-regulated genes (7). Additionally, patients with JDM had increased cell-free mitochondrial DNA in their peripheral blood, with even higher levels noted in patients with calcinosis. Autoantibodies directed against mitochondrial antigens (AMA) were present in 40% of JDM patients and were significantly associated with calcinosis (odds ratio = 6.1). Interestingly, AMA became elevated prior to the clinical diagnosis of calcinosis and may have prognostic potential (8).

The role of neutrophils in JDM is also being explored, though they are not typically found in muscle biopsies. Investigators identified neutrophils in damaged muscle tissue *via* electron microscopy and showed that those neutrophils had phagocytized calcium crystals. The crystals induced neutrophil extracellular traps (NETs), leading to local tissue damage and inflammation. Macrophages were also shown to phagocytize calcium crystals (9).

## Rates and risk factors

Reported rates of calcinosis and associated risk factors vary. Recent North American reviews described calcinosis in 2-5% of patients at JDM diagnosis and in 13-25% overall (10–13). International cohort studies have shown higher rates ranging from 9 to 11% at diagnosis and 20 to 40% developing calcinosis over the disease course (14–21). A multinational, multicenter study of patients with the myositis-specific antibody (MSA) anti-nuclear matrix protein 2 (NXP2) described calcinosis in 15% at diagnosis and 42% overall (22). In other cohorts tested for MSA, rates of calcinosis by MSA were rarely significant, with the exception of one that showed 75% of patients with anti-melanoma differentiation-associated gene 5 (MDA5) antibodies developed calcinosis (16). Risk factors for initial development (and in some cases recurrence) of calcinosis included prolonged duration of disease prior to treatment (10, 12, 14), nailfold abnormalities at baseline (11), chronic disease course (11), lipodystrophy (12), joint contractures (12), longer time to regain full muscle strength (15), higher muscle biopsy score (16), and disease onset prior to age 6 years (16). In one cohort, 77% of calcinosis resolved in an average of 5.6 months, though 25% had recurrence at the original site in an average of 5 years. For those patients whose calcinosis never resolved, 71% had calcinosis at diagnosis and presented with longer disease duration (10). A review of 49 adult patients (median age 24 years) sought to characterize long-term outcomes, and at a median of 11.5 years post diagnosis, 55% had calcinosis. For each additional year of disease, the odds of calcinosis increased 12%, whereas for each additional year of age at diagnosis, the odds of calcinosis decreased 19% (23).

A review from the Childhood Arthritis and Rheumatology Research Alliance (CARRA) registry compared the mean UV index in the month prior to symptom onset in a cohort of over 500 patients. The results showed an association between mean UV index and development of calcinosis, though this was dependent on race; there was a correlation in non-African American patients between higher UV exposure and development of calcinosis (24). In another study from the CARRA registry, African American patients had twofold increased odds of calcinosis compared to other patients (25). These studies suggest there are both genetic and environmental factors in the development of calcinosis.

A variation in presentation of JDM is clinically amyopathic disease (CAJDM). A retrospective review comparing patients with CAJDM to patients with muscle involvement found that none of the CAJDM patients developed calcinosis during a median follow-up of 2.9 years. This was compared to 33% of the JDM patients, suggesting that calcinosis may be a less frequent comorbidity in CAJDM (26).

## Treatment

Treatment guidelines for calcinosis in JDM are largely lacking. In 2017, the SHARE committee (Single Hub and Access point for pediatric Rheumatology in Europe) published treatment recommendations for JDM, but declined to give a specific recommendation for calcinosis due to lack of evidence. The SHARE committee suggested that the following treatments may be beneficial in patients with calcinosis: bisphosphonates, infliximab, abatacept, diltiazem, probenecid, intravenous immunoglobulin (IVIG), intralesional steroids, or surgical resection (27). Similarly, a CARRA 2017 consensus treatment plan for JDM with persistent skin rash excluded those patients with more than “mild” calcinosis (per opinion of the treating physician) (28).

A survey of over 100 pediatric rheumatologists on their current practice of assessing and treating calcinosis in JDM found a variety of immunomodulators are used in clinical practice, the most common being: IVIG, systemic glucocorticoids, methotrexate, mycophenolate mofetil (MMF), and colchicine. By far, the most commonly used alternative agent was bisphosphonates, followed by calcium channel blockers and topical sodium thiosulfate (STS). Experienced physicians, defined as having seen more than 10 cases of calcinosis, were significantly more likely to use these alternative agents compared to physicians who had seen fewer cases (29). A 2017 survey of pediatric rheumatologists found that 52.5% of physicians felt that the use of biologics would reduce calcinosis (30). The current literature regarding treatment is summarized below by medication and medication class.

### Abatacept

Abatacept is a fusion protein of cytotoxic T-lymphocyte antigen-4 and immunoglobulin that inhibits costimulation of T-cell activation. Abatacept is proposed to treat calcinosis through inhibition of macrophages and a subsequent decrease in cytokine production (31). An open-label, non-randomized trial of abatacept in combination with standard therapy recently completed in 10 JDM patients. The primary outcome was the number of patients meeting the International Myositis Assessment and Clinical Studies Group (IMACS) definition of improvement in myositis disease activity at week 24. Study results indicate that 20% had worsening of calcinosis, though final results have not yet been published (ClinicalTrials.gov, identifier NCT02594735) (4). In a retrospective review of four patients initially treated with glucocorticoids, IVIG, methotrexate, and hydroxychloroquine, with two patients also receiving rituximab, calcinosis developed a median of 18.5 months after disease onset. Abatacept led to both clinical and imaging improvement of calcinosis in all patients within 6-12 months (32). Another case report described a 16-year-old diagnosed with JDM at age seven who developed calcinosis after poor medication adherence. Having failed multiple immunomodulators and even surgical resection, she was started on abatacept with clinical improvement after 3 months and no new calcinosis lesions at 1 year (31).

### Bisphosphonates

Bisphosphonates are thought to prevent formation of calcium phosphate crystals, decrease bone turnover, and inhibit macrophage activity (5). A case series of three children used pamidronate to effectively treat calcinosis. The first patient, a 12-year-old boy with a 3-year history of JDM, presented with calcinosis of his finger. He received pamidronate 1 mg/kg/day for three consecutive days every 3 months and experienced complete resolution after 1 year. The other two patients had improvement in, but not resolution of, calcinosis, though patient two had adherence concerns, and patient three had extensive calcinosis following a 1-year delay in diagnosis. All patients were concurrently treated with glucocorticoids and hydroxychloroquine, and two were treated with methotrexate (33). Another case report described a girl who also had a 1-year delay in diagnosis and presented with severe calcinosis in her abdomen and pelvis. She was anti-NXP2 antibody positive and started on glucocorticoids, methotrexate, cyclophosphamide, and monthly pamidronate infusions. Two years later, plain radiographs showed near resolution of calcinosis (34).

### JAK inhibitors

The purported mechanism of Janus kinase (JAK) inhibitors in calcinosis is a decrease of ROS-mediated mitochondrial calcium accumulation (5). One case report detailed a 5-year-old boy lost to follow-up for 8 months, who returned with extensive calcinosis leading to joint contractures and inability to ambulate. After failing multiple immunomodulators, baricitinib was added and regression of lesions was observed after 6 months (35). Another case report described a young boy diagnosed at age 2.5 years, who developed calcinosis 6 months later. After failing cyclophosphamide, azathioprine, MMF, infliximab, adalimumab, rituximab, tacrolimus, cyclosporine, and IVIG, he was started on baricitinib for progressive calcinosis. After 6 months of treatment, no new calcinotic lesions appeared (36). In contrast, two patients with refractory JDM who failed 3-6 other immunomodulatory medications were treated with baricitinib for 24 weeks without improvement (37).

Two case reports describe noteworthy response to tofacitinib. The first patient was a 7-year-old boy, anti-NXP2 antibody positive, who developed calcinosis 2 years after diagnosis, and subsequently failed therapy with cyclophosphamide, a calcium-channel blocker, pamidronate, MMF, and infliximab. The second patient was a 9-year-old girl who presented with calcinosis at diagnosis, but failed to improve with IVIG, pamidronate, MMF, infliximab, and rituximab. Three months after starting tofacitinib, patient one had resolution of calcinosis and patient two had 50% improvement by physical exam and X-ray findings (38).

In contrast, another case report described a 13-year-old girl with anti-NXP2 antibody-positive JDM who had improvement of overall disease activity, but no change in calcinosis after 12 months of ruxolitinib (39).

### Mycophenolate mofetil

Mycophenolate mofetil is an antimetabolic agent that inhibits B- and T-cell proliferation by blocking DNA synthesis. One case report described a 5-year-old girl who developed calcinosis universalis 3 months after discontinuing cyclosporine, despite remaining on glucocorticoids and methotrexate. She was started on pamidronate (1 mg/kg/dose on three consecutive days every 3 months), colchicine, and IVIG without improvement. One year later, methotrexate was transitioned to MMF, pamidronate was stopped, and resolution of all calcinosis was noted within 6 months. She remained without calcinosis on MMF, glucocorticoids, and colchicine 3 years later (40).

### Rituximab

Rituximab is a monoclonal antibody targeting CD20 on B-cells, leading to B-cell depletion (5). Aggarwal et al. published a randomized, placebo-phase-controlled trial of rituximab for cutaneous manifestations in a cohort of refractory adult DM and JDM patients. Subjects were randomized to either rituximab early at week 0/1 or rituximab late at week 8/9. At baseline, 46% of the 48 JDM patients were noted to have calcinosis, and at 44 weeks, 57%. Though improvements were noted in cutaneous disease activity, no major change was noted in calcinosis (41). In contrast, a case series of four patients on rituximab showed resolution of calcinosis in one patient, and improvement in existing lesions with no new lesions in three patients. Though all patients were receiving concomitant glucocorticoids and other immunomodulators, no changes had been made in the 6 months prior to rituximab, suggesting rituximab may have been beneficial (42). Finally, a case report of a 6-year-old who presented with calcinosis approximately 1 year after JDM diagnosis, saw near resolution of calcinosis after 8 months of rituximab. The patient had previously failed the combination of pamidronate and etanercept (43).

### Sodium thiosulfate

Sodium thiosulfate is a calcium chelating agent (5). There is currently an ongoing single-arm, open-label trial of intravenous (IV) STS to treat JDM-related calcinosis, with the primary endpoint being change in calcinosis activity visual analog scale score pre- and post-treatment. The study will include patients who are at least 7-years-old (ClinicalTrials.gov, identifier NCT03267277) (4).

One case report of IV STS described an 11-year-old boy diagnosed with JDM at age two, who presented with calcinosis at age nine and failed both pamidronate and diltiazem. He was started on daily IV STS with improvement in pain, but adverse effects limited treatment and no improvement in calcinosis was seen after 6 months (44). Similarly, a woman in her 20s with a 13-year history of JDM and a 10-year history of extensive calcinosis, who had already failed diltiazem, bisphosphonates, and cinacalcet specifically for calcinosis, developed side effects of fatigue and nausea and had progression of her calcinosis while on IV STS (45).

A case report of topical STS used for a 15-year-old with amyopathic dermatomyositis described improvement in size and number of lesions after 8 weeks. The largest lesions were first surgically removed (46).

### TNF inhibitors

The proposed mechanism of tumor necrosis factor (TNF) inhibitors (TNFi) in treating calcinosis is suppression of cytokine release (5). In a retrospective analysis of 60 children with refractory JDM, of which 47% had calcinosis, complete resolution of calcinosis was noted in 29% of patients treated with infliximab or adalimumab, with an additional 54% noting improvement. At time of TNFi initiation, 80% were also on methotrexate, azathioprine, or hydroxychloroquine, and 68% on oral glucocorticoids. The median time to improvement was 2.75 years, with a range of 3 months to 10 years, highlighting that prolonged treatment may be necessary in some patients (47).

Another case report described an anti-NXP2 antibody-positive 10-year-old girl with persistent disease activity over 3 years despite glucocorticoids, IVIG, methotrexate, cyclosporine, thalidomide, colchicine, MMF, and bisphosphonates. The patient was subsequently started on adalimumab (continued on methotrexate, colchicine, and glucocorticoid), with symptomatic improvement within 3 months and a decrease in calcinosis after 1 year (48). A similar report of an anti-NXP2 antibody-positive 4-year-old with 3 years of continuous disease activity despite glucocorticoids, IVIG, methotrexate, and cyclosporine, described significant improvement in calcinosis 9 months after starting infliximab, thalidomide, and aluminum hydroxide (49).

### Tocilizumab

Tocilizumab is a monoclonal antibody that binds the IL-6 receptor (30). In a case report of a patient with refractory, anti-NXP2 antibody-positive JDM complicated by calcinosis, who developed nodular regenerative hyperplasia of the liver, regression of calcinosis was noted after the patient was transitioned to tocilizumab and IVIG to avoid hepatotoxic medications (50).

### Other therapies

Carbon dioxide laser therapy has numerous applications within dermatology (51) and was shown in a case report of a 22-year-old with JDM to improve calcinosis lesion size and clinical symptoms after just one treatment (52). Additionally, a 33-year-old woman with a 7-year history of refractory calcinosis noted profound improvement in her calcinosis within days of receiving a tattoo, mimicking treatment *via* microneedling. She then received inkless tattoo of her entire left lower extremity and interestingly saw improvement in bilateral lower extremities. No other new interventions were added during this time (53).

## Discussion

Despite a growing body of literature, knowledge gaps remain in our understanding of the pathogenesis, risk factors, and most effective treatments for calcinosis in JDM. In a review of 563 patients with JDM from the CARRA registry, 39% of patient global activity scores were at least 2 points different than the corresponding physician global activity scores. Among these patients, 10% had calcinosis, and in multivariable analysis, calcinosis was an independent predictor of discordance in patient and provider global disease activity scores. This finding suggests that calcinosis has important impacts on the perception of JDM disease activity for both physicians and patients and should possibly be a research priority (54).

The lack of clear treatment guidelines is the result of many factors, including the rarity of JDM and the variability in reported rates of JDM-related calcinosis. A survey of practicing pediatric rheumatologists indicated that only 17% had treated more than 20 cases (29). Another factor is that most of the current literature is limited to anecdotal case reports and case series, which make it difficult to differentiate correlation and causation. The case reports and case series are also highly variable in underlying therapies, duration of treatment, duration of follow-up, and ultimately outcomes. For example, though high dose glucocorticoids have been the mainstay of treatment for JDM, and thus the prevention of calcinosis, Orandi et al. reported a retrospective review of 31 patients treated with lower dose and shorter duration glucocorticoids. The incidence and prevalence of calcinosis in the cohort was comparable to published literature, suggesting such high dose glucocorticoids may not be required to prevent sequelae like calcinosis (55).

Though there have been randomized controlled trials (RCTs) conducted in adult patients with DM, adult and juvenile DM are known to have key variations in presentation, complications, and outcomes, and so findings from these studies cannot be directly applied to pediatric patients (56). Additionally, true comparative studies are difficult without better objective measures of calcinosis; the majority of the current literature defines a presence or absence of calcinosis, making it difficult to quantify improvement on a given therapy.

Additional areas of future research should include the phenotype and specificity of MSA profiles. A recent case study described three patients, all with seronegative polyarthritis, who tested positive for anti-NXP2 antibodies, suggesting that they may not be specific for dermatomyositis (57). Additionally, a case series of patients with anti-NXP2 antibodies reported four patients who tested positive for multiple MSA, including anti-MDA5 and anti-Mi2 (22). While a false positive is certainly possible, further investigation is warranted.

Ultimately, despite the advancement in understanding of certain risk factors for calcinosis in JDM, particularly that early and aggressive treatment can help prevent development, questions about best treatment options remain. While RCTs are considered the most robust, they may not be feasible given the rarity of JDM and JDM-related calcinosis. Future research with more innovative comparative effectiveness approaches and standardized assessment tools for calcinosis are needed to define the most efficacious diagnostic and treatment for calcinosis in JDM.

## Author contributions

EW, LC, and CP: conceptualization. CP: investigation and writing—original draft preparation. CP, LC, JD, and EW: writing—review and editing. All authors read and agreed to the published version of the manuscript.
